# Enhanced anti-tumor effects of the PD-1/PD-L1 blockade by combining a highly absorptive form of NF-kB/STAT3 inhibitor curcumin

**DOI:** 10.1186/2051-1426-2-S3-P210

**Published:** 2014-11-06

**Authors:** Taeko Hayakawa, Juri Sugiyama, Tomonori Yaguchi, Atsushi Imaizumi, Yutaka Kawakami

**Affiliations:** 1 Division of Cellular Signaling, Institute for Advanced Medical Research, Keio University School of Medicine, Tokyo, Japan; 2 Science Group, THERAVALUES Corporation, Tokyo, Japan

## 

The PD-1/PD-L1 blockade is now recognized as one of the basic immune interventions for development of combination cancer immunotherapy. We have been screening chemical libraries to obtain compounds which have an activity to augment anti-tumor activity of the PD-1/PD-L1 blockade. We have previously shown that activation of NF-kB and STAT3 signals in both cancer cells and immune cells plays important roles in cancer cell-induced immunosuppression, and their inhibition may augment anti-tumor CTL. Curcumin, a major active component of turmeric, has activities to inhibit both NF-kB and STAT3 signals. We have recently developed a highly absorptive form of curcumin which oral administration resulted in high plasma concentration. In this study, we evaluated combination treatment of this highly absorptive form of curcumin with the PD-1/PD-L1 blockade. Curcumin inhibited significantly *in vitro *production of IL6 and IL8 by human ovarian cancer cell lines (OC) having activated NF-kB and STAT3 signaling. In nude mice implanted with human OC, T-cell stimulatory activity of murine DC was impaired partly through increased human IL-6 from human OC. Systemic *p.o*. administration of curcumin decreased human IL6 in mouse serum and restored T-cell stimulatory activity of murine DC in spleens and tumors. In two syngeneic murine colon cancer models, NF-kB dependent IL6 producing MC38 in B6 mice and NF-kB non-activated IL6 non-producing CT26 in Balb/C mice, curcumin *p.o*. administration resulted in the significant augmentation of tumor antigen-specific CD8+ T-cell induction accompanied by increased T-cell stimulatory activity of DC via decreased STAT3 and NF-kB signaling, although curcumin did not inhibit proliferation of both tumor cell lines. Furthermore, *p.o*. administration of curcumin into non-tumor bearing B6 mice immunized with ovalbumin (OVA) peptide augmented OVA-specific CTL induction. These results indicate that curcumin not only enhances tumor antigen specific T-cell via reversal of tumor-induced immunosuppression (e.g. IL6 induced DC impairment), but also enhances CTL via directly acting on immune cells. Since PD-1 positive T cells were infiltrated in tumors expressing PD-L1, we evaluated combination of curcumin and anti-PD-L1 blockade, and found that combination of curcumin and anti-PD-L1 Ab had synergistic anti-tumor activity. These results indicate that combination of curcumin which enhances induction of tumor antigen specific, PD-1 positive CTL in tumors via acting on both cancer cells and immune cells and local PD-1/PD-L1 blockade, is an attractive strategy for development of effective cancer immunotherapy.

